# The Association Between Theory of Mind, Psychopathic Traits, Borderline Personality Traits, and Severity of Substance Use Disorder in Women: A Comparative Analysis

**DOI:** 10.31083/AP44175

**Published:** 2025-06-24

**Authors:** Sercan Karabulut, Seyhan Uzar Uçkun

**Affiliations:** ^1^Department of Psychiatry, School of Medicine, Akdeniz University, 07070 Antalya, Türkiye; ^2^Centre of Alcohol and Substance Addiction Treatment, Ataturk State Hospital, 07192 Antalya, Türkiye

**Keywords:** Theory of Mind, substance use disorder, borderline personality disorder, psychopathic personality

## Abstract

**Objective:**

Studies investigating social cognition impairments in substance use disorders (SUD) emerged from attempts to understand the influence of social interactions on substance use. This study aimed to measure Theory of Mind (ToM) performance and possible interactions between ToM performance, personality traits, and substance use severity.

**Methods:**

Participants (n = 153) were assessed using the Reading the Mind in the Eyes, Dokuz Eylul Theory of Mind Index, Addiction Profile Index (API), Borderline Personality Questionnaire (BPQ), Basic Empathy Scale (BES), and Levenson Self-Report Psychopathy scale (LSRP).

**Results:**

Cluster analysis identified two groups: a ‘high ToM’ (n = 59, 41.2%) and a ‘low ToM’ (n = 84, 58.8%) group. Comparative analysis showed that the API effect of substance use on life subscale scores (*p* = 0.033), BES total (*p* = 0.003), and affective empathy subscale scores (*p* = 0.001) were higher in the high ToM group compared with the low ToM group. Conversely, BPQ impulsivity subscale scores (*p* = 0.011), LSRP total (*p* = 0.026), and primary psychopathy subscale scores (*p* = 0.007) were lower in the high ToM group compared with the low ToM groups. Binary logistic regression analysis showed that lower affective empathy scores (odds ratio (OR) = 0.896, 95% confidence interval (CI) (0.818–0.982), *p* = 0.019) and higher primary psychopathy scores (OR = 1.099, 95% CI (1.011–1.195), *p* = 0.027) predicted ToM abilities in women with SUD.

**Conclusions:**

This study provides novel evidence that in women with SUD, affective psychopathic traits and lack of affective empathy predict lower ToM abilities. These findings suggest that intervention targeting affect-related psychopathy dimensions may be effective in alleviating ToM disabilities.

## Main Points

1. Substance use severity scores in women with substance use disorder did not show any difference between the low Theory of Mind ability and high Theory of Mind ability groups.

2. Affective psychopathic traits and affective empathy levels were found to be associated with Theory of Mind impairments in women with substance use disorder.

3. Borderline personality traits did not predict Theory of Mind impairment level in women with substance use disorder.

## 1. Introduction

Substance use disorder (SUD) is a mental health condition marked by the persistent and problematic use of substances despite harmful outcomes [1]. Individuals with SUD often experience difficulties in social cognition, which can lead to significant interpersonal problems and challenges in social adjustment [2, 3]. Recent theories suggest that deficits in social cognition—particularly in recognizing facial emotions, identifying postures, and distinguishing between one’s own and others’ emotions—play a key role in understanding these challenges [4, 5, 6]. A systematic review on Theory of Mind (ToM) impairments has indicated that these sociocognitive deficits may be linked to the adverse social and interpersonal consequences seen in SUD [7].

ToM, the capacity to infer others’ thoughts and feelings, is essential for accurately predicting and interpreting behavior in various settings. Impairments in ToM have been documented among individuals with schizophrenia, bipolar disorder, and autism spectrum disorders [8, 9, 10]. An increasing amount of research is investigating the connection between ToM impairments and substance use. For example, studies utilizing the Reading the Mind in the Eyes Task (RMET) found that methamphetamine users and individuals with opioid use disorder scored lower than healthy controls [11, 12, 13].

Further research using narrative-based tasks, such as the Faux Pas Recognition Task and the Movie for the Assessment of Social Cognition, has shown that individuals with SUD perform worse on ToM tasks compared with non-SUD controls [12, 14]. The overlap between ToM impairments and executive function deficits suggests that social cognition challenges may play an essential role in the neural circuitry underlying addiction [13, 15, 16].

Studies have also highlighted a close association between ToM and cognitive empathy, which involves consciously interpreting others’ mental states [17, 18, 19]. In contrast, emotional empathy refers to experiencing another’s emotions, whether real or inferred [20]. Evidence shows that individuals with SUD often demonstrate deficits in both cognitive and emotional empathy, suggesting that lower empathy levels may be a developmental risk factor for substance related behaviors [21, 22].

Additionally, personality disorders—antisocial personality disorder (ASPD) and borderline personality disorder (BPD)—commonly coincide with SUD and are linked to sociocognitive impairments [23]. A recent meta-analysis reported that individuals with BPD show poorer performance on cognitive ToM tasks compared with affective ToM tasks [23]. Another study found that people with BPD struggle with recognizing emotions and intentions, possibly marking this as a distinctive endophenotypic trait [24]. Moreover, psychopathic traits, such as impulsivity, emotional dysregulation, and a tendency toward antisocial behavior, are also associated with deficits in both cognitive and affective ToM [25]. These findings imply that similar sociocognitive deficits may be shared between SUD and personality disorders, potentially stemming from common developmental processes [26, 27].

Furthermore, research on sociocognitive impairments in women with SUD is limited, although women with SUD often present with complex clinical profiles involving psychiatric comorbidities, social stigma, parenting responsibilities, and family pressures [14, 28, 29, 30, 31]. Women are also more vulnerable to the effects of interpersonal problems on craving and relapse compared with men with SUD [32], highlighting the need for further research specifically focused on their sociocognitive impairments [33, 34].

This study aims to explore social cognitive abilities through the RMET and cognitive ToM tasks, examine addiction severity using the Addiction Profile Index, and assess empathy, borderline traits, and psychopathic traits through self-report measures. Specifically, this study seeks to:

1. Determine whether there is significant ToM impairment among a female sample with SUD.

2. Investigate whether ToM impairments correlate with varying levels of substance use severity.

3. Examine whether there are significant differences in borderline and psychopathic traits among groups categorized by ToM impairments.

## 2. Materials and Methods

### 2.1 Participants and Procedures

Participants were recruited from the outpatient addiction treatment clinic (OTC) at Antalya Atatürk State Hospital with eligible participants being women consecutively admitted between September, 2021 and March, 2023. Inclusion criteria required participants to be female, aged 17–65 years, to have used at least one substance, meet SUD criteria, be literate, and be able to complete assessments in Turkish. Exclusion criteria included comorbid alcohol use disorder, primary psychotic disorders, intellectual disability, or unwillingness to participate. Additionally, those reporting severe suicidal or homicidal ideation during interviews were excluded. The final sample included 153 women diagnosed with at least one SUD. All participants were assessed using the Structured Clinical Interview for Diagnostic and Statistical Manual of Mental Disorders (DSM-5)-Clinician Version (SCID-5-CV) for SUDs, excluding tobacco products [1, 35]. Eligible participants completed face-to-face semi-structured questionnaires, self-report paper-based scales, and clinical tests administered by a researcher.

The study received approval from the Ethics Committee of Antalya Research and Training Hospital (approval number 8/13) and was conducted in accordance with ethical principles (World Medical Association’s Declaration of Helsinki). Written informed consent was obtained from all participants.

### 2.2 Measures

#### 2.2.1 Sociodemographic and Clinical Variables

The questionnaire was divided into two sections: The first part gathered individual data, including age, housing, relationship status, income, employment, education, and history of imprisonment or parole/probation. The second part focused on clinical variables, capturing information on tobacco use frequency and quantity, alcohol use frequency, screenings for infectious diseases, history of suicide attempts, intravenous substance use and equipment sharing, risky sexual behaviors, motives for initial substance use, age at first substance use, family history of substance use, and the number of substances used in the past year.

#### 2.2.2 Self-Report Scales

Borderline personality traits were evaluated using the Borderline Personality Questionnaire (BPQ), a true/false measure with nine subscales aligned with DSM-5 criteria for borderline personality disorder: impulsivity, affective instability, fear of abandonment, unstable relationships, self-image issues, self-harm, feelings of emptiness, intense anger, and quasi-psychotic states. The BPQ’s total score exhibited high internal consistency (Cronbach’s alpha = 0.94) in its original study, with subscales ranging from 0.65 to 0.84 [36, 37]. In this study, the BPQ showed good internal consistency, with a total score alpha of 0.87 and subscale alphas between 0.85 and 0.87.

The Levenson Self-Report Psychopathy scale (LSRP) was used to measure psychopathy-related personality traits and behaviors. This 26-item, 4-point Likert-type scale captures both primary psychopathy (callous/manipulative traits) and secondary psychopathy (behavioral problems). The primary psychopathy subscale had an alpha of 0.82, whereas secondary psychopathy had an alpha of 0.63 in the original study [38, 39]. In our sample, the LSRP total score alpha was 0.84, with subscale alphas of 0.74 for primary and 0.90 for secondary psychopathy.

Empathy levels were measured with the Basic Empathy Scale (BES), which uses a 5-point Likert scale. This 20-item measure includes 11 items for affective empathy (Cronbach’s alpha = 0.79) and nine for cognitive empathy (Cronbach’s alpha = 0.85) [40, 41]. In this study, the BES demonstrated high internal consistency, with an overall alpha of 0.85 and subscale alphas of 0.79 for affective empathy and 0.88 for cognitive empathy.

#### 2.2.3 Clinician-Administered Scales

The Addiction Profile Index (API) was employed to measure addiction severity. The API consists of 37 items across five subscales, each rated by the clinician on a 3-point scale. These subscales assess substance use characteristics, dependency diagnosis, impact of substance use on life, cravings, and motivation for quitting. The original API total score alpha was 0.89, with subscale alphas from 0.63 to 0.86 [42]. In this study, the API’s total score alpha was 0.75, with subscale alphas from 0.62 to 0.77.

To diagnose SUD, the SCID-5-CV was used. Released in 2014, this clinician-administered semi-structured interview includes 10 modules covering 39 of the most common clinical diagnoses and allows for screening of 16 additional disorders per DSM-5 criteria [1, 35].

#### 2.2.4 Theory of Mind Tasks

The Reading the Mind in the Eyes Task (RMET) was used to evaluate the affective aspect of ToM. RMET comprises 36 photographs showing only the eye region of individuals expressing various emotions or intentions. Participants choose one of four words that best describe the emotion or intent displayed [11]. The Turkish version of the RMET, validated by Yıldırım *et al*. [43], excludes some unreliable items, resulting in a 32-item version used in this study. Scores ranged from 0 to 32, with lower scores indicating poorer ToM abilities. The original RMET had a Cronbach’s alpha of 0.72, while our sample showed 0.74.

The Dokuz Eylul Theory of Mind Index (DEToMI) was used to measure cognitive ToM skills. This test includes seven stories with 24 questions, combining open-ended and forced-choice formats, and assesses skills in understanding first- and second-order false beliefs, metaphor and irony comprehension, and faux pas recognition. Developed and validated by Değirmencioğlu* et al*. [44], it was initially tested in patients with schizophrenia and control groups. The maximum score is 18, with higher scores reflecting greater ToM ability. The original reliability coefficient for DEToMI factor items was 0.66, while our sample’s was 0.63.

### 2.3 Statistical Analyses

An initial descriptive analysis was conducted. Although the sample consisted of 153 participants, the data of 10 participants were excluded from the analysis due to missing critical values. Statistical analyses were performed on 143 participants’ data.

Second, a cluster analysis was performed to discriminate homogenous subgroups within the main sample. The two-step algorithm’s automatic clustering function was used to identify the number of clusters and Schwarz’s Bayesian Criterion (BIC) was used to calculate the ratio of distances between the clusters. According to the literature and clinical observations, we planned to perform this analysis on DEToMI scores, RME scores, level of schooling, and age [11, 43]. Although education levels were reported to be highly associated with ToM abilities, cluster analysis with four variables resulted in two homogenous subgroups that did not differ significantly according to DEToMI and RME scores; we decided to exclude level of schooling from the analysis. The age variable was also excluded from the analysis due to nonsignificant group structures. In the final model, cluster analysis was applied with two variables (DEToMI scores and RME scores). Cluster analysis yielded two groups: a low ToM group and a high ToM group. Pearson’s Chi-squared test, Fisher’s Exact test, and the Fisher-Freeman-Halton test were used to examine the differences between categorical variables. Normality was assessed using the Kolmogorov-Smirnov and Shapiro-Wilk tests. The independent sample *t*-test was used for normally distributed continuous variables. To examine the relationship between clinical variables and ToM abilities, binary logistic regression analysis was used. The pre-defined level of significance was *p* < 0.05. All analyses were conducted using Statistical Package for the Social Sciences (SPSS) version 27.0 (IBM Corporation, Armonk, NY, USA).

## 3. Results

### 3.1 Characteristics of the Sample Demographics

The majority of participants were in the ‘20–29’ age subgroup (n = 85, 59.4%), 27 (18.9%) were uninsured, 107 (74.8%) had a low income, and 108 (75.5%) were unemployed. In terms of education level, most of the group were in the ‘6–9 years’ of schooling group (n = 67, 46.9%). In terms of relationship, 56 (39.2%) were separated/divorced, whereas 49 (34.3%) were single. The most frequent diagnosis was multiple substance use disorder (37.8%) followed by opiate use disorder (37.1%) and stimulant use disorder (19.5%). For nearly half the group, their first experience of substance use was motivated by succumbing to peer pressure (45.3%). The most frequently experienced first use was of cannabis (51%). Table 1 shows the sociodemographic characteristics of the sample.

**Table 1.** **Sociodemographic characteristics of women with substance use disorder**.

Sociodemographics		n = 143 (%)	Sociodemographics		n = 143 (%)
Age (years)			History of Imprisonment		
	<20	16.1		None	31.5
	20–29	59.4		Yes	68.5
	30–39	20.3	History of Parole/Probation		
	>40	4.2		None	42
Insurance Status				Yes	58
	Insured	81.1	Alcohol Use		
	Uninsured	18.9		None	62.9
Income Status				Social drinker	33.6
	Low-Income (<8500 TL)	74.8		Regular drinker	3.5
	Moderate-High Income (>8500 TL)	25.2	Persons who Inject Drugs		
Employment				Yes	32.2
	Unemployed	75.5		None	67.8
	Temporary/part-time	7.5	Contagious Diseases		
	Full-time	15.4		Hepatitis C Virus	12.7
	Student	1.6		Syphilis	5.6
Years of schooling				Hepatitis B Virus	2.8
	5 years	12.5		Human Immunodeficiency Virus	1.4
	6–9 years	46.9		HCV+syphilis	2.8
	10–13 years	32.2		HIV+HCV+syphilis	0.7
	>14 years	8.4	Risky Sexual Behavior		
Relationship				Yes	33.6
	Single	34.3		None	66.4
	Widowed/separated	39.2	Motive of First Drug Experience		
	In a relationship	26.5		Peer pressure	45.3
Diagnosis				Novelty seeking	22.3
	Multiple Substance Use Disorder	37.8		History of family use	14.1
	Opiate Use Disorder	37.1		Psychiatric symptoms	18.3
	Stimulant Use Disorder	19.5	Family History of Substance Use		
	Cannabis Use Disorder	2.8		None	53.8
	Other	2.8		Yes	46.2

$1.00 USD = 38.63 TL. HIV, human immunodeficiency virus; HCV, hepatitis C virus; TL, Turkish Lira.

### 3.2 Description of Clusters

**Fig. 1.**
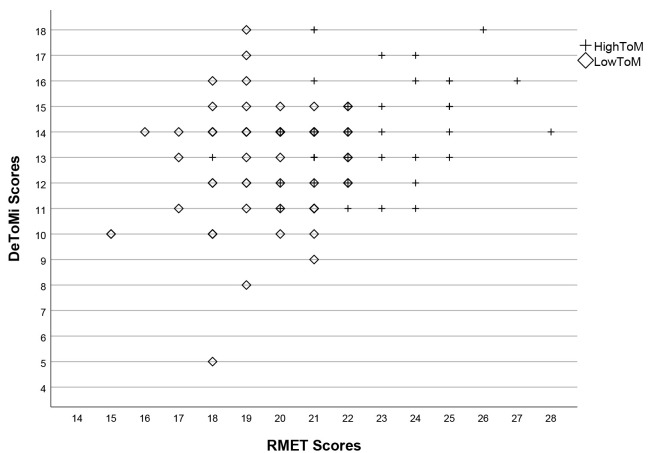
**Scatter plot illustrating Theory of Mind Scores in the high and low Theory of Mind groups**. DeToMi, Dokuz Eylul Theory of Mind Index; RMET, Reading the Mind in the Eyes Test.

Cluster analysis yielded a two-cluster solution with the largest ratio of distances (Schwarz’s BIC = 181.507, Ratio of Distance Measures = 1.179). The first cluster we labeled the ‘high ToM’ group because the group members’ mean DEToMI and Reading the Mind in the Eyes (RME) scores were higher than the second cluster members’ mean DEToMI and RME scores. This group represented 41.2% of the sample (n = 59). The second cluster we labeled the ‘low ToM’ group. It consisted of 84 participants (58.8%) whose ToM task scores were lower than the first group’s scores. Table 2 shows the composition of the two clusters by ToM scores. Fig. 1 shows a histogram displaying the ToM Scores in terms of the two clusters.

**Table 2.** **Composition of the two clusters by Theory of Mind scores**.

Cluster Label	n (%)	Reading the Mind in the Eyes Test Score (mean ± SD)	Dokuz Eylul Theory of Mind Index Score (mean ± SD)
High ToM Scores	59 (41.2)	22.64 ± 1.57	14.07 ± 1.40
Low ToM Scores	84 (58.8)	19.62 ± 1.22	13.39 ± 1.71
*p*		<0.001	0.013

SD, standard Deviation; ToM, theory of mind.

### 3.3 Sociodemographic Differences Between Cluster Members

The Chi-squared analysis results showed that there were no differences between cluster groups in ‘living with’ status (*p* = 0.827), schooling (*p* = 0.606), income status (*p* = 0.058), relationship status (*p* = 0.510), or employment (*p* = 0.309). Compared with the high ToM group, members of the low ToM group were more likely to have a suicide attempt history (64.3% vs 47.5%, *p* = 0.045).

### 3.4 Clinical Differences Between Cluster Members

In terms of IV substance use and risky sexual behaviors, no differences were found among the groups (*p* = 0.722, *p* = 0.516, respectively). More than half of the high ToM group started to use substance under peer-pressure, although the difference was not statistically significant (52.5%, *p* = 0.06).

The *t*-test results showed that the number of substances used within last year and age of first substance experience were not different between the groups (*p* = 0.556, *p* = 0.985, respectively).

Borderline personality traits were compared by BPQ and subscales between the two clusters. Members of the low ToM cluster had higher impulsivity subscale scores compared with high ToM cluster members (*p* = 0.011, Cohen’s d = 0.56). Other subscales and total BPQ scores did not differ between the groups.

Participants with low ToM scores had higher primary psychopathy scores and total LSRP scores than participants with high ToM scores (*p* = 0.007, Cohen’s d = 0.59; *p* = 0.026, Cohen’s d = 0.48, respectively). Comparison of empathy scores showed that total BES and affective empathy, but not the cognitive empathy subscale, scores were significantly higher in the high ToM group among clusters (*p* = 0.003, Cohen’s d = 0.66; *p* = 0.001, Cohen’s d = 0.71; *p* = 0.118; respectively). Table 3 shows the clinical differences between the two clusters.

**Table 3.** **Comparison of clinical scales between the high ToM and low ToM groups**.

	High ToM	Low ToM	*p*
	mean ± SD	mean ± SD	
API total score	6.47 ± 1.53	6.28 ± 1.37	0.453
API dependency diagnosis subscale score	8.54 ± 2.88	8.34 ± 2.82	0.684
API substance use subscale score	1.03 ± 0.98	1.11 ± 0.82	0.625
API the effect of substance use on the person’s life subscale score	14.88 ± 4.14	13.44 ± 3.80	0.033
API craving subscale score	4.38 ± 2.23	4.47 ± 2.10	0.814
API motivation for quitting using substances subscale score	5.74 ± 1.15	5.77 ± 0.66	0.854
BPQ Impulsivity subscale score	3.61 ± 1.62	4.52 ± 1.60	0.011
BPQ Self-image subscale score	3.98 ± 2.52	4.66 ± 2.54	0.217
BPQ Abandonment subscale score	5.61 ± 2.32	5.80 ± 2.48	0.723
BPQ Emptiness subscale score	5.83 ± 2.58	5.91 ± 2.22	0.879
BPQ Affective instability subscale score	6.22 ± 2.21	6.55 ± 2.51	0.527
BPQ Unstable relationships subscale score	5.59 ± 1.77	5.75 ± 1.34	0.630
BPQ Intense anger subscale score	5.95 ± 3.05	6.89 ± 2.77	0.143
BPQ Self-mutilation subscale score	3.07 ± 2.21	3.98 ± 2.09	0.057
BPQ Quasi-psychotic states subscale score	2.83 ± 2.22	3.41 ± 2.21	0.232
BPQ total score	42.68 ± 14.57	47.45 ± 14.42	0.133
LSRP Primary Psychopathy subscale score	28.09 ± 7.32	32.47 ± 7.50	0.007
LSRP Secondary Psychopathy subscale score	28.07 ± 5.11	28.86 ± 6.03	0.507
LSRP total score	56.16 ± 10.41	61.33 ± 10.93	0.026
BES Cognitive Empathy subscale score	35.66 ± 5.19	33.74 ± 5.85	0.118
BES Affective Empathy subscale score	41.00 ± 5.71	36.28 ± 7.27	0.001
BES total score	76.66 ± 9.00	70.02 ± 10.70	0.003

API, Addiction Profile Index; BPQ, Borderline Personality Questionnaire; LSRP, Levenson Self-Report Psychopathy scale; BES, Basic Empathy Scale.

### 3.5 Substance Use Severity Differences Between Cluster Members

The results showed that the characteristics of substance use, craving, dependence diagnosis, and motivation subscale scores were not significantly different among clusters. The effect of substance use on the person’s life subscale scores were significantly higher in the high ToM group compared with the low ToM group (*p* = 0.033, Cohen’s d = 0.36). Total API scores analysis showed that high ToM cluster members had higher substance severity than low ToM cluster members, although the difference was not statistically significant (*p* = 0.453). Table 3 shows substance use severity differences between clusters.

### 3.6 Logistic Regression Analysis Results

A logistic regression analysis including independent variables associated with ToM disabilities was performed. The *p* value of the model was determined as 0.005, and the multivariate model explained the response variable well with Cox & Snell R^2^ (0.261) and Nagelkerke R^2^ (0.349) values. As a result of the Hosmer-Lemeshow test, the predicted significance level for the H-L test statistical value (*p* = 0.217) and the model fit very well. As a result of the model, a significant relationship was found between ToM abilities and LSRP Primary Psychopathy subscale score (Odds Ratio (OR) = 1.099 (95% Confidence Interval (CI) 1.011–1.195), *p* = 0.027) and BES Affective Empathy subscale score (Odds Ration = 0.896 (95% Confidence Interval 0.818–0.982), *p* = 0.019). Table 4 shows the logistic regression analysis results.

**Table 4.** **Logistic regression analysis of factors associated with ToM abilities**.

Variables		OR (95% CI)	*p*
Age (years)		0.996 (0.902–1.010)	0.936
Education Level			
	University (reference)		
	High School (9–12 years)	0.192 (0.025–1.467)	0.112
	Middle School (5–8 years)	0.576 (0.086–3.841)	0.569
	Primary School (4 years)	0.429 (0.031–5.848)	0.525
API the effect of substance use on the person’s life subscale score		0.942 (0.802–1.106)	0.465
BPQ Impulsivity subscale score		1.417 (0.937–2.144)	0.098
BPQ Self-mutilation subscale score		1.041 (0.774–1.400)	0.789
LSRP Primary Psychopathy subscale score		1.099 (1.011–1.195)	0.027
BES Affective Empathy subscale score		0.896 (0.818–0.982)	0.019

OR, odds ratio; CI, Confidence Interval.

## 4. Discussion

This study examined differences in addiction severity, borderline and psychopathic traits, and empathy among women with SUD, with respect to ToM scores. The most notable findings were: (i) substance use severity did not differ between groups of women based on ToM abilities, and (ii) women with higher psychopathic traits and lower empathy levels exhibited lower ToM abilities, independent of borderline traits. These results suggest that addressing psychopathic traits through targeted therapeutic interventions may help improve ToM impairments in women with SUD.

The link between ToM functioning and alcohol use has been consistently documented [6, 45]. However, when analyzing the results according to specific substances, the findings are mixed. Several studies have reported that patients with opioid use disorder, maintained on buprenorphine or methadone, performed worse on ToM tasks compared with those in abstinence [15, 46, 47]. In contrast, a study on poly-substance users found no abnormalities in a video-based social cognition assessment [48]. Although many studies indicate significant differences in ToM scores between patients and healthy controls, our findings suggest that substance use severity may not be the primary factor influencing the ability to predict others’ mental states in individuals with SUD.

BPD is characterized by affective, cognitive, behavioral, and interpersonal symptoms, with emotional instability, impulsivity, and unstable relationships being central features. Although the frequent co-occurrence of BPD and SUD, along with shared risk factors, suggests that ToM impairments may be present in both disorders, the literature shows inconsistent results. For example, Harari *et al*. [49] reported cognitive and affective ToM impairments in BPD patients, but subsequent studies failed to replicate these findings [50, 51]. A meta-analysis concluded that there was no significant difference in ToM impairments between BPD patients and healthy controls [23], with the authors attributing the variability in results to multifactorial causes. In line with these findings, our study found no significant differences in borderline personality traits between the high and low ToM groups [23].

A systematic review of psychopathic traits and ToM found a negative correlation between psychopathy and ToM task performance (pooled r = –0.126), indicating that higher psychopathic traits were associated with a reduced ability to understand others’ thoughts, feelings, intentions, and beliefs [25]. Previous studies also found that the interpersonal/affective traits of psychopathy had a greater effect size than the lifestyle/antisocial traits. Interpersonal/affective components are thought to represent the core of emotional and empathic deficits associated with psychopathy, leading to stronger correlations with ToM impairments compared with lifestyle/antisocial traits [25, 52]. In our study, scores on the primary psychopathy subscale, which measures emotional affect, were higher than those on the secondary psychopathy subscale. Additionally, the affective component of the BES was lower in the low ToM group.

This study has several limitations. First, the sample included only women with SUD, which may limit the generalizability of the findings to men. Second, the clusters were not perfectly matched in terms of education levels, although these differences were not statistically significant. Third, we did not group participants by substance type, as doing so would have reduced the sample size, making it harder to draw meaningful conclusions. Fourth, we did not include a control group of non-substance users. Fifth, although the results showed significant difference between the two groups, the DEToMI results had considerable overlap across groups. Studies conducted with healthy controls have reported larger differences than our study, thus lack of a control group might explain the relative homogeneity in our sample [44, 53]. Finally, we did not account for the potential effects of psychotropic medications, which could be an important limitation. Larger studies with control groups are needed to determine whether these findings are specific to women with SUD or applicable to other clinical groups. There is evidence that cognitive flexibility impacts social cognition [12], and further research in female populations may help inform better therapeutic interventions and sociocognitive rehabilitation strategies [7].

## 5. Conclusions

In summary, we provide novel evidence that in women with SUD, affective psychopathic traits and lack of affective empathy predict lower ToM abilities. It was affective empathy, rather than cognitive empathy, that differentiated the low and high ToM groups. The capacity to emotionally resonate with others (affective empathy) is central to primary psychopathy. Conversely, individuals with psychopathy do not exhibit deficits in cognitive empathy; they possess the ability to understand others’ perspectives. This cognitive skill may, in fact, facilitate their exploitation and manipulation of others, as they are not hindered by empathic emotions. The results also showed that ToM abilities were independent of the severity of substance use.

These findings suggest that interventions (treatment and rehabilitation strategies) targeting affect-related psychopathy dimensions may be effective in alleviating ToM disabilities.

## Data Availability

The data that support the findings of this study are available from the corresponding author upon reasonable request.
